# 基于智能手机的便携式毛细管电泳装置检测消毒剂中2种季铵盐

**DOI:** 10.3724/SP.J.1123.2021.04030

**Published:** 2021-11-08

**Authors:** Yuanyu WANG, Ruihua ZHANG, Qiang ZHANG, Chengxi CAO, Liuyin FAN, Weiwen LIU

**Affiliations:** 1.上海交通大学电子信息与电气工程学院, 上海 200240; 1. School of Electronic Information & Electrical Engineering, Shanghai Jiao Tong University, Shanghai,200240, China; 2.上海交通大学学生创新中心, 上海 200240; 2. Student Innovation Center, Shanghai Jiao Tong University, Shanghai, 200240, China

**Keywords:** 毛细管电泳, 电容耦合式非接触电导检测, 基于智能手机的便携式装置, 季铵盐, 消毒剂, capillary electrophoresis (CE), capacitively coupled contactless conductivity detection (C^4^D), smartphone-based portable device, quaternary ammonium salts (QAs), disinfector

## Abstract

现有的小型毛细管电泳(CE)装置仍采用平板或计算机进行数据处理和分析,其便携性仍存在明显不足。针对这一问题,发展了一种基于智能手机的CE装置,实现了真正的便携式定量分析。该装置集成了电容耦合式非接触式电导检测(C^4^D)和蓝牙通信功能,并提供了手机界面软件。通过手机界面软件,不仅可以控制CE装置的电泳运行,还可以实时接收C^4^D检测器发出的数据信息,显示电泳图谱和进行数据处理。该装置尺寸为20 cm×20 cm×15 cm,重量为2 kg。为了验证所设计装置的性能,采用季铵盐(QAs)消毒剂(十二烷基二甲基苄基溴化铵(DDBAB)和十二烷基三甲基溴化铵(DTAB))作为分析对象。实验数据表明,DDBAB和DTAB线性范围分别为20~1000和30~1000 μmol/L,线性回归方程的相关系数(*R*^2^)分别为0.9995和0.9989,检出限(LOD)分别为10和13 μmol/L,日内相对标准偏差(RSD, *n*=3)分别为1.9%和2.7%。另外实验对DDBAB和DTAB混合离子液进行了测试,在8 min内可实现基线分离。最后,对现场使用的新洁尔灭消毒液中QAs进行了加标回收试验,DDBAB和DTAB的回收率分别为100.5%~101.5%和96.2%~99.3%。研究结果表明,所开发CE装置具有线性好、LOD低、重复好、准确性高,尤其便携好等优点,可用于消毒液中QAs现场定量检测。

## Abstract (en)

The existing miniature capillary electrophoresis (CE) devices use a tablet or a computer for data processing and analysis, which hinders their portability. In order to solve this problem, a smartphone-based CE device was proposed, which allowed for real portable quantitative analysis. The device integrated the functions of capacitively coupled contactless conductivity detection (C^4^D) and Bluetooth communication. Furthermore, a Kotlin language-based application with a user-friendly interface was developed. The application could not only control the electrophoresis run in the CE device but also receive the data from the C^4^D detector in real time, display the electrophoretogram, and process the data. The peak areas could be calculated automatically on the smartphone, and the migration time could be obtained. The size of the developed device was 20 cm×20 cm×15 cm, and its weight was 2 kg. Quaternary ammonium salts (QAs) in disinfectors (dodecyl dimethyl benzyl ammonium bromide (DDBAB) and dodecyl trimethyl ammonium bromide (DTAB)) were used as the analytes to verify the performance of the developed device. The experimental data showed that the linear ranges of DDBAB and DTAB were from 20 to 1000 and from 30 to 1000 μmol/L, respectively. The correlation coefficients (*R*^2^) of DDBAB and DTAB were 0.9995 and 0.9989, respectively, indicating good linearity between the peak area and concentration. The limits of detection (LODs) of DDBAB and DTAB were 10 and 13 μmol/L, respectively. The intra-day relative standard deviations (RSDs, *n*=3) of DDBAB and DTAB were 1.9% and 2.7% respectively, revealing good repeatability. In addition, a mixture of DDBAB and DTAB was tested. Two QAs were separated within 8 min, showing good selectivity. Finally, QAs in a bromo geramine disinfector used in the field were tested to further validate the performance of the designed device. The recoveries of DDBAB and DTAB were 100.5%-101.5% and 96.2%-99.3%, respectively, indicating good accuracy. The developed device has the advantages of good linearity, low LOD, good repeatability, high accuracy, and real portability, and it can be used for the quantitative detection of QAs in disinfectors.

季铵盐(QAs)作为一种阳离子表面活性剂^[1]^,是洗涤剂^[2]^、化妆品^[3]^和建筑材料防腐剂^[4]^中的重要化工原料,同时作为常见消毒剂的主要成分被广泛应用^[5,6,7]^。这类QAs消毒剂的效能主要受浓度、接触时间和pH值等环境因素的影响。若QAs消毒剂浓度无法达到要求,则会大大降低消毒效果;同时,QAs消毒剂具有较强的毒性,对其过量使用又会威胁到人体健康。因此,现场检测QAs消毒剂具有重要意义。

常见的实验室检测QAs含量的方法有紫外分光光度法^[8]^、高效液相色谱法^[9]^、月桂基硫酸钠滴定法^[10]^和毛细管电泳法(CE)^[11]^。这几种方法具有精密度高、检出限(LOD)低、检测速度快等优点,然而都需要在特定的实验室环境中进行,不易做到便携式和现场检测。CE与电容耦合非接触式电导检测(CE-C^4^D)样品消耗少,LOD低,灵敏度高,检测速度快,为CE的便携式设计带来了新的思路^[12,13,14]^。

为了实现CE-C^4^D便携化,众多研究者做了相关研究^[15]^。2001年,Kappes等^[16]^首次提出了一款便携式CE-C^4^D装置,其尺寸为34 cm×17.5 cm×17.5 cm,重7.5 kg。Mai等^[17]^于2013年发展了一种便携CE-C^4^D检测装置,该装置放置在一个45 cm×35 cm×15 cm的手提箱中,总重8 kg。2014年,Nguyen等^[18]^研制了一款便携式CE-C^4^D装置,其尺寸为40 cm×28 cm×21 cm,重6 kg。次年,Gregus等^[19]^设计了一种基于C^4^D的CE装置,其尺寸为20 cm×33 cm×17 cm,重量不足5 kg。2021年,Graf等^[20]^提出了一种基于电容数字转换技术的CE-C^4^D原理样机,但没有报道装置的尺寸和重量。虽然近年来报道的便携式CE-C^4^D装置无论是在体积还是重量上,其便携性都有很大改善,但它们都采用计算机或平板电脑进行数据处理和分析,因此在便携化上仍然存在明显不足。

针对上述问题,本文发展了一种基于智能手机的CE-C^4^D装置,实现了真正的便携式现场分析。该装置集成了C^4^D和蓝牙通信功能,并提供了手机界面软件。通过手机界面软件,不仅可以控制CE装置的电泳运行,还可以实时接收C^4^D检测器发出的数据信息,显示电泳图谱和进行数据处理。为了验证装置的性能,对QAs消毒剂中的十二烷基二甲基苄基溴化铵(DDBAB)和十二烷基三甲基溴化铵(DTAB)进行了定量检测,并针对现场使用的新洁尔灭消毒液进行了加标回收试验。研究结果表明,所开发的装置具有线性好、LOD低、重复好、准确性高,尤其便携好等优点,可用于消毒液中QAs现场定量检测。

## 1 实验部分

### 1.1 仪器

ET120 C^4^D传感器检测头(澳大利亚eDAQ公司);聚酰亚胺涂层熔融石英毛细管(总长50 cm,有效长度35 cm,内径75 μm,外径365 μm,中国河北永年光纤厂);高压电源(-15 kV,中国天津恒博高压电源厂);超纯水系统(德国SG Water公司)用来生产电导率低至0.055 μS/cm的去离子水;pH计(瑞士Mettler Toledo公司)。

### 1.2 试剂

氢氧化钠(NaOH,纯度98%)、盐酸(HCl,质量分数36.0%~38.0%)等试剂均购自上海化学试剂公司;乳酸(Lac,纯度≥80%)、DDBAB(纯度98%)购自上海泰坦科技股份有限公司;*β*-丙氨酸(*β*-Ala,纯度98%)购自中国医药集团有限公司;DTAB (纯度98%)购自上海麦瑞尔化学技术有限公司;新洁尔灭消毒液购自上海培婕医疗仪器专营店。

### 1.3 背景液与样品液的制备

分别取Lac 2.815 g、*β*-Ala 2.2273 g,置于1 L容量瓶中,用去离子水定容,得到浓度为25 mmol/L的Lac-*β*-Ala缓冲液^[21]^,用pH计测得缓冲液的pH为3.6,放入试剂瓶中备用。

分别称取DDBAB 3.845 g、DTAB 3.083 g,置于1 L容量瓶中,用去离子水定容,放置在试剂瓶中备用。实验时,用配制好的Lac-*β*-Ala背景缓冲液将样品液分别稀释至1000、500、250、125、67.5、33.75、16.875 μmol/L,作为标准样品液。

取购买的新洁尔灭消毒液2.5 mL,现场喷洒到滤纸上并回收,回收的滤纸通过去离子水缓慢冲洗,将冲洗后的溶液用去离子水定容至25 mL,使用时再用背景缓冲液稀释10倍作为实际样品液。

### 1.4 毛细管的预处理及实验条件

在使用前先将毛细管依次用1.0 mol/L NaOH、去离子水和1.0 mol/L HCl分别冲洗20、10和20 min,最后用Lac-*β*-Ala背景缓冲液冲洗30 min,等待毛细管内部环境稳定后进行实验。

实验控制在25 ℃室温下进行,设置电泳分离电压为-15 kV,在毛细管阳极端采用高度差10 cm的静水压进样,进样时间为5 s。

### 1.5 基于智能手机的CE-C^4^D装置

基于智能手机的便携式CE-C^4^D装置高压电源与检测模块见图1a,手机控制器见图1b。其中,检测模块包括C^4^D传感器和C^4^D检测电路,而C^4^D检测电路由信号处理模块、微控制单元(MCU)和蓝牙模块组成。在图1a中,CE信号经C^4^D传感器采集,之后由信号处理模块对检测信号进行基线调节、放大和模数转换并送入MCU, MCU处理后将数据信息输出给蓝牙模块并由其传递给手机控制器。手机控制器接收到数据信息后,在屏幕上实时绘制出电泳图谱。另外,用户还可通过手机界面软件发送控制信息,经信号检测模块中的蓝牙模块接收后由集成在MCU内的数模转换器(DAC)转换为模拟电压信号以控制-15 kV高压电源的输出,从而控制电泳运行。

**图1:**
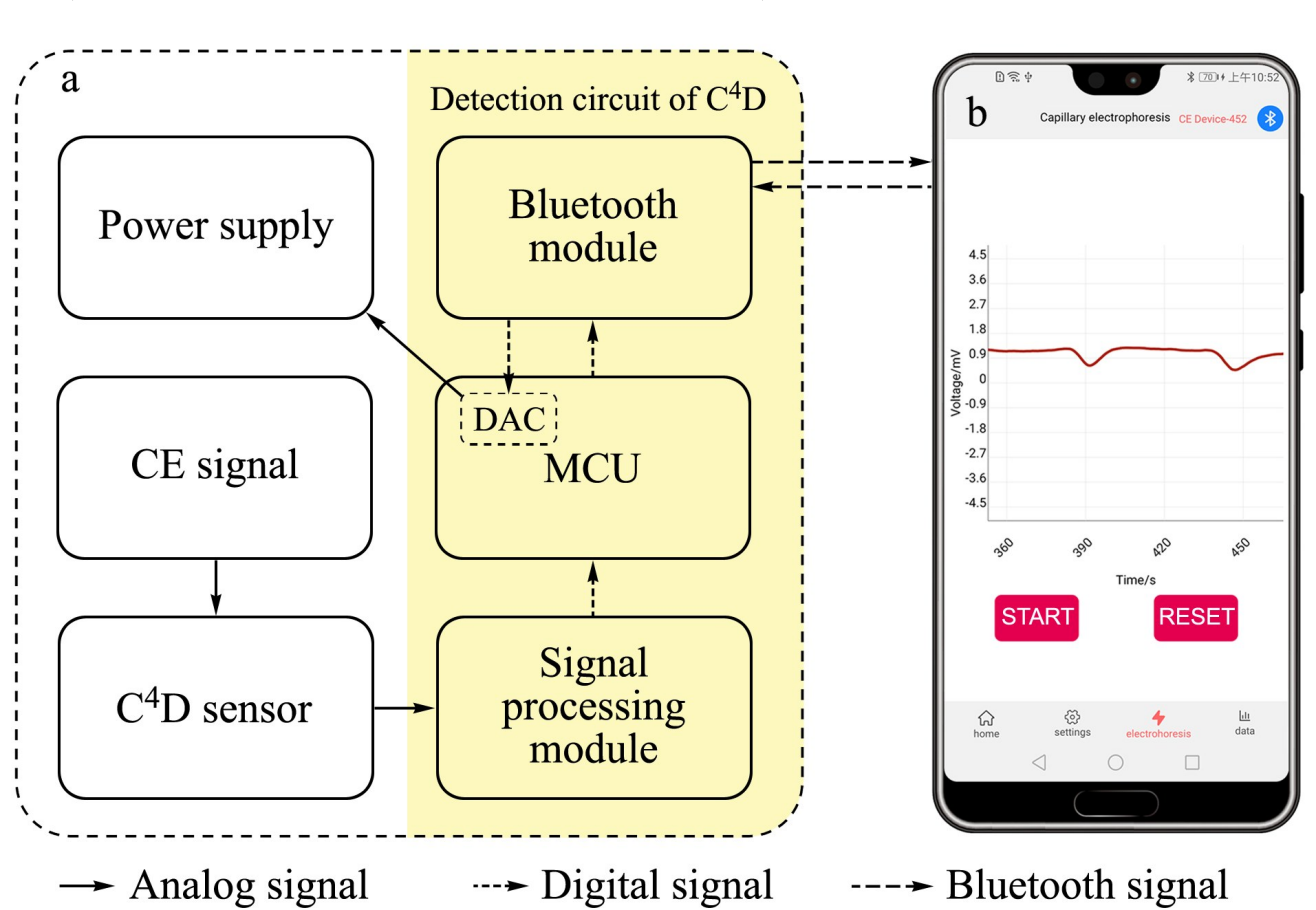
基于智能手机的便携式CE-C^4^D装置(a)高压电源 与检测模块和(b)手机控制器

手机界面软件是基于Kotlin语言开发的一款Android应用程序,其主要包括蓝牙连接模块、数据展示模块以及结果分析模块。蓝牙连接模块负责蓝牙的连接管理,使用过程中,手机应用程序端与检测电路端通过各自的蓝牙模块建立连接进行通信,相应的通信函数会收发信息并根据信息种类进行处理。信息分为控制信息与数据信息,这两种信息在软件编码中采用不同的编码字段来进行识别。数据展示模块主要实现了手机端的电泳图谱可视化,利用开源图表库E-Chart在手机界面中内嵌了绘图控件,手机端接收到数据信息后,在绘图控件上实时绘制出电泳图谱。检测结束后,采集的数据会保存在应用程序缓存中并传入结果分析模块。结果分析模块由数据优化、数据计算以及数据保存3部分组成。其中数据优化部分采用小波变换对接收到的数据信息进行基线校准和去噪。数据计算部分通过寻峰与积分函数计算出结果峰的迁移时间与面积并进行定量分析。数据保存部分可对数据进行保存,方便用户共享与进一步分析。

开发的便携式CE-C^4^D装置如图2所示,装置尺寸为20 cm×20 cm×15 cm,重量为2 kg,主要分为分离和检测两部分。分离部分为标准的CE结构,包括毛细管a、被放置在装置中间并用金属屏蔽的高压电源模块b、分别设置在高压电源两侧的阳极背景液池c与阴极背景液池d和一个样品液池e。检测部分包括放置在毛细管a进样端35 cm位置处的C^4^D传感器f和自制的C^4^D检测电路。其中C^4^D检测电路内置有蓝牙模块,可与智能手机进行无线通信。

**图2:**
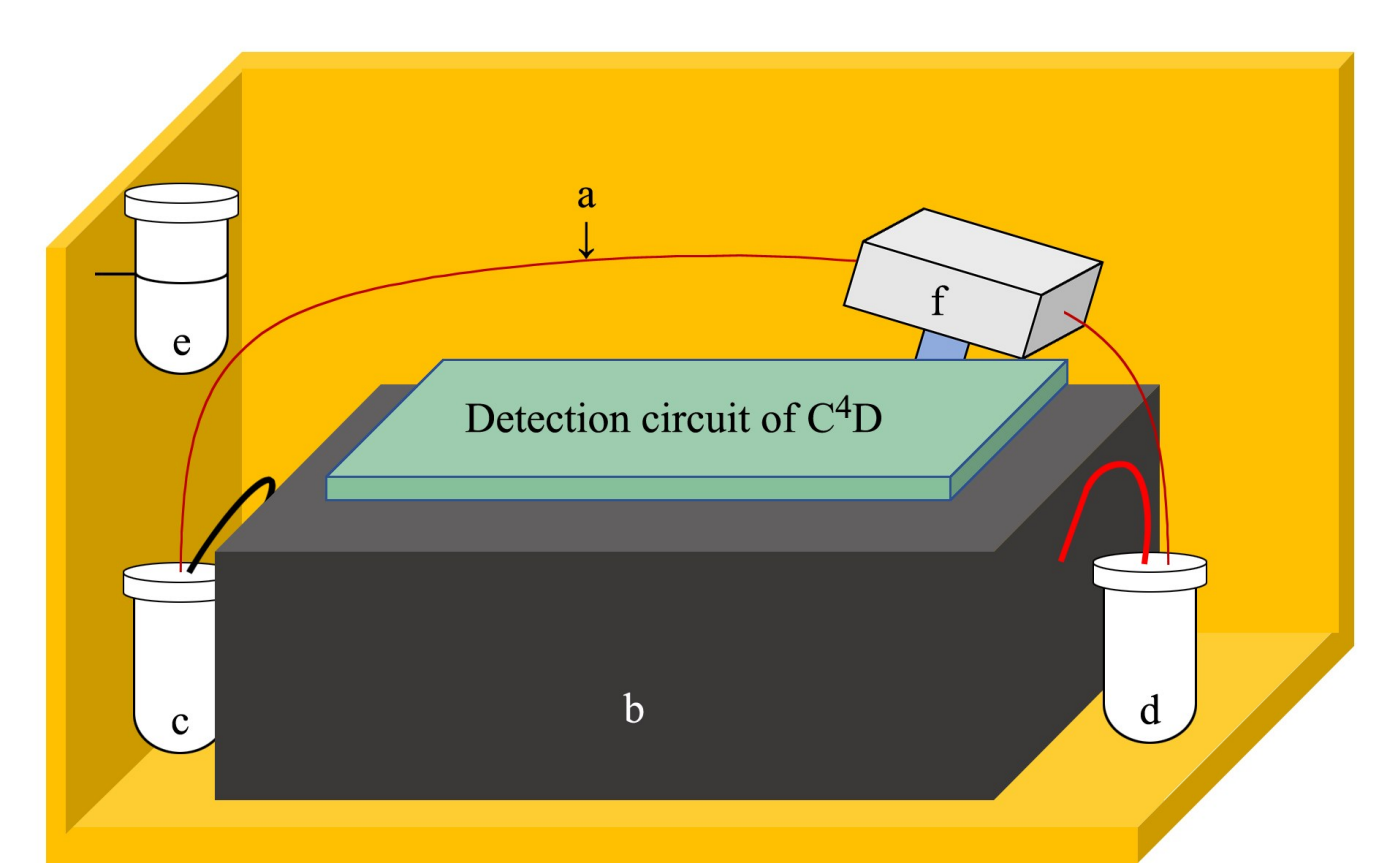
便携式CE-C^4^D装置示意图

### 1.6 电泳过程与定量计算方法

实验时,将手机控制器与便携式CE-C^4^D装置通过蓝牙连接。然后在上述实验条件下对预处理后的毛细管进行进样,待毛细管放回阳极背景液池5 s后在手机控制器上启动电泳运行。手机控制器随后接收数据信息并在屏幕上实时绘制出电泳图谱,10 min后电泳过程自动停止。

定量计算方法:先使用便携式CE-C^4^D装置对5种已知浓度的标准溶液进行检测,手机控制器接收到数据信息后通过寻峰与积分函数计算出峰面积,利用最小二乘法拟合出峰面积与浓度的标准曲线并将曲线参数保存在手机中。在实际样品检测时,同样通过寻峰与积分函数自动寻峰并计算出峰面积,峰面积将自动代入建立好的标准曲线中计算出其对应的浓度,从而实现定量计算。

## 2 结果与讨论

### 2.1 装置性能验证

取1.3节配制的8种标准样品液,在1 d内重复测量3次,计算后得到两种QAs的线性方程、相关系数、线性范围、检出限及相对标准偏差,如表1所示。从表中可以看出,DDBAB与DTAB的线性范围分别为20~1000和30~1000 μmol/L,相关系数(*R*^2^)分别为0.9995和0.9989,相对标准偏差分别≤1.9%和≤2.7%,验证了装置对于DDBAB与DTAB的检测具有良好的线性和可重复性。最后逐步稀释标准样品液进行测试,观察电泳图谱直到图谱中目标峰高与基线噪声幅度比值为3倍(*S/N*=3)时,记录此时对应的浓度,得到DDBAB与DTAB的LOD分别为10和13 μmol/L(见表1)。

**表1:** 两种季铵盐的线性方程、相关系数、线性范围、检出限及相对标准偏差

Compound	Regression equation	R^2^	Linear range/(μmol/L)	LOD/(μmol/L)	RSD (n=3)/%
DDBAB	Y=6.2478X+0.0057	0.9995	20-1000	10	≤1.9
DTAB	Y=5.3290X-0.0940	0.9989	30-1000	13	≤2.7

DDBAB: dodecyl dimethyl benzyl ammonium bromide; DTAB: dodecyl trimethyl ammonium bromide; *Y*: peak area; *X*: concentration, μmol/L.

分别对25 mmol/LLac-*β*-Ala背景液(见图3a)、1 mmol/LDDBAB单离子液(见图3b)、1 mmol/LDTAB单离子液(见图3c)以及1 mmol/LDDBAB和DTAB混合离子液(见图3d)进行测试。DDBAB和DTAB在8 min内完全基线分离,保留时间分别为450 s和390 s,而且峰形良好,这也验证了装置对于QAs消毒剂具有良好的选择性。

**图3:**
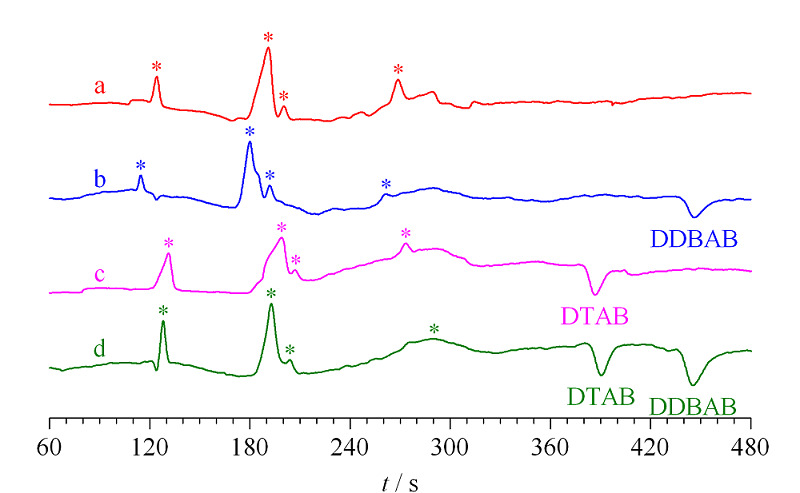
(a)背景液、(b)DDBAB、(c)DTAB及其(d)混合 离子溶液的电泳图谱

### 2.2 实际样品分析

利用开发的装置对1.3节制备的实际样品液从上午9:00开始至晚上9:00结束,每隔4 h进行一次测,结果如图4所示。图中4条曲线基本保持一致,其中系统峰受到人为操作的影响,峰面积差异较大,但其出峰时间基本相同。此外,450 s时,4个时间点DDBAB峰面积的RSD为2.3%,出峰时间的RSD为1.1%,结果表明方法有较好的可重复性。

**图4:**
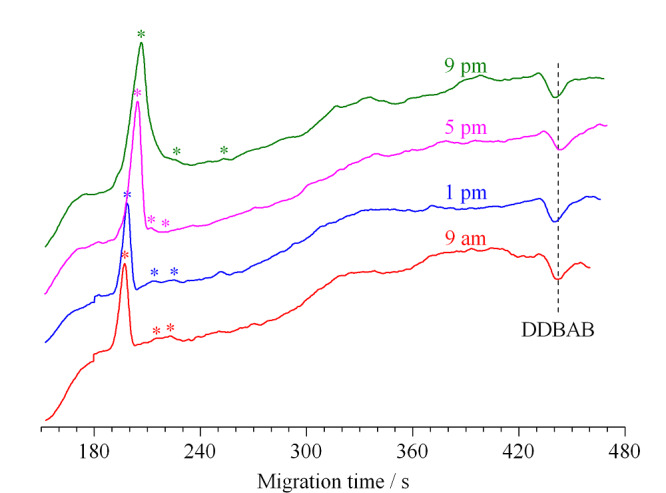
新洁尔灭消毒液中QAs的重复性测试

新洁尔灭消毒液实际样品的主要有效成分为DDBAB,在现场回收的新洁尔灭消毒液中分别加入0.8、1.0和1.2 mg的DDBAB。同时为了考察不同QAs在实际样品中的检测效果,回收后的样品液中还分别额外添加了0.8、1.0和1.2 mg的DTAB。随后,分别取加标后的样品液用去离子水稀释20倍再用背景液稀释10倍后进行实验,结果如表2所示。DDBAB和DTAB的回收率分别为100.5%~101.5%和96.2%~99.3%。实验结果显示,样品液的有效成分为DDBAB,且不同QAs能在样品中有效基线分离,表明了开发的装置能应用于不同QAs的分离分析。

**表2:** 新洁尔灭消毒液中DDBAB与DTAB的 加标回收率(*n*=5)

Compound	Background/ mg	Spiked/ mg	Found/ mg	Recovery/ %	RSD/ %
DDBAB	1.07	0.8	1.882	101.5	1.47
		1	2.081	100.5	2.13
		1.2	2.286	101.3	1.19
DTAB	0	0.8	0.794	99.3	1.17
		1	0.962	96.2	1.35
		1.2	1.187	98.9	2.29

### 2.3 与其他方法比较

将本文开发的CE-C^4^D装置与现有便携式CE-C^4^D装置进行比较,结果见表3。从表中可知,本文介绍的装置由于采用了手机替代电脑进行数据处理和分析,减小了装置体积,在现场检测以及便携性上有独特优势。然而,装置在LOD上还有进一步的提升空间。

**表3:** 本文开发的CE-C^4^D装置与现有装置的比较

Device	LOD/ (μmol/ L)	Size/ (cm×cm ×cm)	Smartphone controlled	Ref.
Automated CE-C^4^D	5	45×35×15	no	[17]
CE-C^4^D of small volumes	0.36	20×33×17	no	[19]
biological fluid				
CDC-based CE-C^4^D	12	not reported	no	[20]
Smartphone-based	10	20×20×15	yes	this
CE-C^4^D				work

CDC: capacitance-to-digital conversion.

## 3 结论

本文发展了一种基于智能手机的便携式CE-C^4^D装置,开发了手机界面软件,通过手机界面软件,不仅可以控制CE装置的电泳运行,还可以实时接收C^4^D检测器发出的数据信息,显示电泳图谱和进行数据处理和分析,进一步提高了检测装置的便携性。为验证该装置的性能,选择两种QAs作为分析物进行了测试。实验结果表明,该装置具有线性好、LOD低、重复好、准确性高,尤其便携好等优点,实现了对QAs消毒剂的定量检测。表明开发的装置可在QAs消毒剂的现场定量检测中发挥重要作用。
